# Supplementary material for: A bacterial endophyte exploits chemotropism of a fungal pathogen for plant colonization
Source: Nat Commun. 2020 Oct 16;11:5264. doi: 10.1038/s41467-020-18994-5 (PMC7567819; doi:10.1038/s41467-020-18994-5)
Supplement: Supplementary file 1 — Supplementary Information [file 41467_2020_18994_MOESM1_ESM.pdf]

Supplementary Information

A bacterial endophyte exploits chemotropism of a fungal pathogen for plant colonization

Palmieri *et al*

Supplementary Table 1. Oligonucleotide primers used in this study.

Primer	Sequence 5'→ 3'	Use
Kanf	GTGCGCGGAACCCCTATTTG	Kan^r cassette
Kanr	TTCTACGGGGTCTGACGCTC	
Gcd1	GTAACGCCAGGGTTTTCCCAGTCACGACGACGCGCCGATCAGTACAACG	*gcd* and *flic* gene knockout constructs
Gcd2	GAGCGTCAGACCCCGTAGAAAGAAGCGTAGCCCATGTTGCG	
Gcd3	CAAATAGGGGTTCCGCGCACTTCGTGTCTAAACTGATCCCAC	
Gcd4	GCGGATAACAATTTCACACAGGAAACAGCTGTGGTGATTTACGAACCCGC	
Flic1	GTAACGCCAGGGTTTTCCCAGTCACGACGGCCGGTGGATGAGTTATATC	
Flic2	GAGCGTCAGACCCCGTAGAAGAGTGTATTCCTTCAAATCTG	
Flic3	CAAATAGGGGTTCCGCGCACCGCAGTTCGGTTACAAACAG	
Flic4	GCGGATAACAATTTCACACAGGAAACAGCCCTTGTTTTGATTCCCGCTG	
PRS426f	GTAACGCCAGGGTTTTCCCAGTCACGACG	
PRS426r	GCGGATAACAATTTCACACAGGAAACAGC	
Gcd5	CTGATGACTTATCACGGCG	*gcd*$^-$ and *flic*$^-$mutant confirmation
Gcd6	CGGGCCCTTTGTGGAAATTC	
Gcd7	CCTGTGGCGCGGGTGATTG	
Gcd8	GCTCCATAGGGTTGCCAGG	
Flic5	CTGATGGTGTTGCTTTGACG	
Flic6	CAATAGGCTGGTCCATCTTG	
Flic7	CAGATTTGAAGGAATACACTC	
Flic8	CTGTTTGTAACCGAACTGCG	
GADPH1	CCAAAAACAGTAACAGCCTTC	Real-time qPCR (Tomato)
GADPH2	TGATGTTGAACTCGTCGCAG	
ACT2	GAGGCACCGCTCTCGTCG	Real-time qPCR (*Fol*)
ACTQ6	GGAGATCCAGACTGCCGCTCA	
Gcd9	GACGAGTTCAGCCTGATGAC	Real-time qPCR (*Ra36*)
Gcd10	GTCGAAACTCACGGTGCTGT	

Supplementary Figures

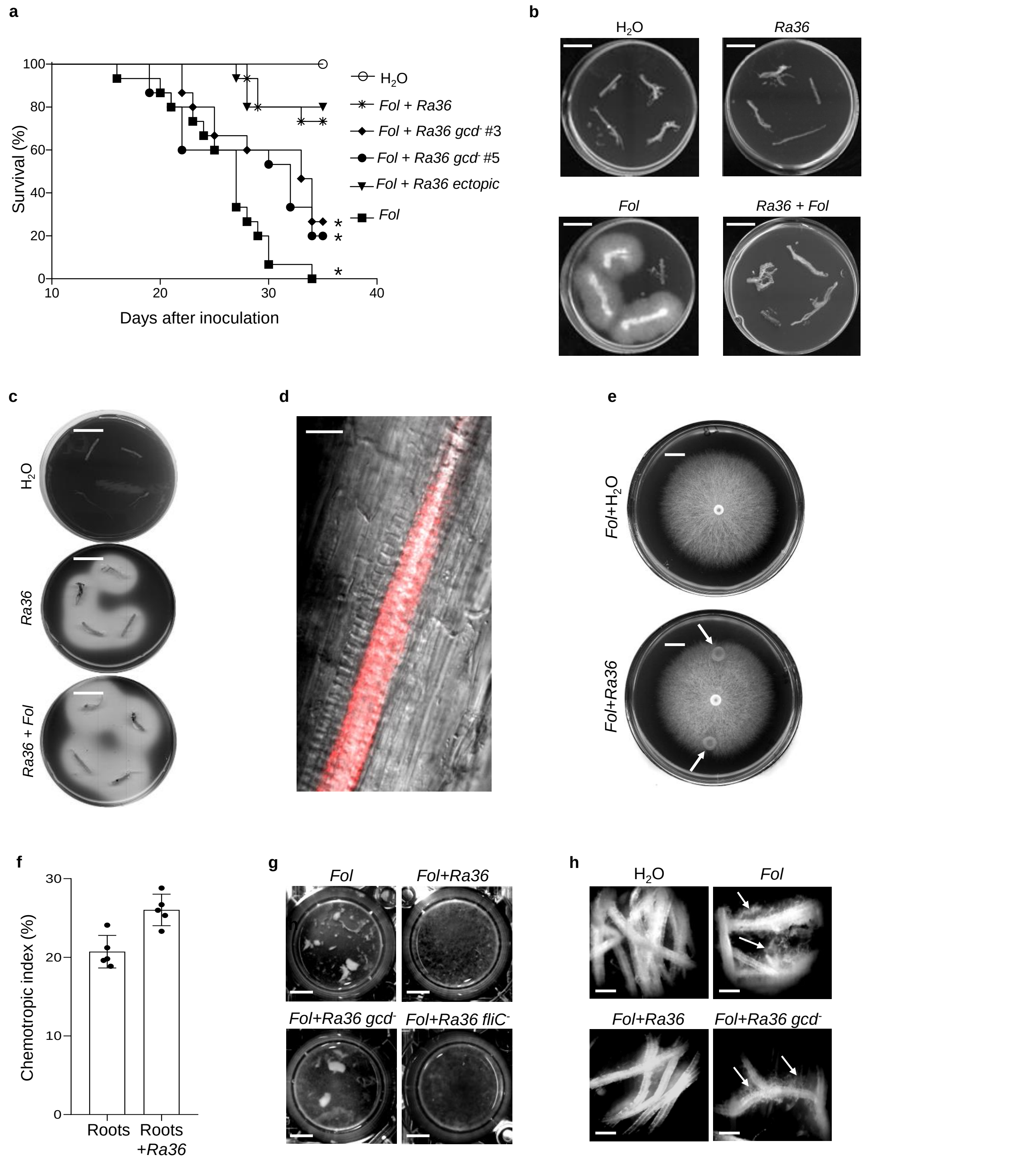

Supplementary Figure 1. *R. aquatilis* inhibits *F. oxysporum* virulence-related functions. a. Kaplan-Meier plot showing survival of tomato plants grown in vermiculite and dip-inoculated or not (H_2O) with *Fol*, the indicated *Ra36* strains or *Fol+Ra36*. Number of independent experiments ($n_{i.ex.}$) = 3; 15 plants/treatment. Data shown are from one representative experiment. $^*P < 0.05$ versus *Fol* + *Ra36* according to log-rank test. *Ra36 gcd*$^-$ #3 and #5 correspond to independent isogenic *gcd*$^-$ deletion mutants. **b, c**. Representative images of plates showing the presence of *Fol* and *Ra36* in tomato plants, 2 weeks after inoculation. Surface-sterilized sections of roots and stems were placed on MMU plates without (**b**) or with the pH indicator bromocresol purple (**c**). Outgrowth of *Fol* or *Ra36* was imaged 5 d after plating. Presence of a clear acid halo in (**c**) is indicative of *Ra36* outgrowth. Number of independent experiments ($n_{i.ex.}$) = 3, with 3 plates each. Scale bar, 2 cm. **d**. Fluorescence microscopy of RFP-tagged *Ra36* inside a tomato root, 4 days after inoculation. Note the presence of *Ra36* in the xylem. $n_{i.ex.}$ = 3, with 2 replicates each. Scale bar, 50 µm. **e**. *Ra36* does not affect colony growth of *Fol*. Fungal microconidia and *Ra36* cells or water as a control (H_2O) were spot-inoculated at 30 mm distance on MMU plates. Colonies were imaged after 4 days. Arrows indicate *Ra36* inoculation spots. $n_{i.ex.}$ = 3, with 3 plates each. Scale bar, 1 cm. **f**. Directed growth of *Fol* germ tubes after 13 h exposure to a gradient of root exudates collected from uninoculated (Roots) or *Ra36*-inoculated (Roots+*Ra36*) tomato plants. $n_{i.ex}$ = 5, with 5 independent batches of cells per treatment; n = 100 cells per batch. Data are

presented as mean values +/- SD from five independent experiments. **g**. Hyphal aggregates of *Fol* after 48 h of growth in liquid MMU medium in the absence or presence of the indicated *Ra36* strain. Cultures were vortexed to dissociate weakly adhering hyphae, transferred to a multiwell plate and imaged in a binocular microscope. $n_{i.ex.}$ = 3, with 4 wells per treatment. Scale bar, 0.2 cm. **h**. Roots of tomato seedlings, either uninoculated or inoculated with the indicated *Ra36* strains, were immersed for 72 h in PDB medium diluted 1:10 in water, in the presence or absence of *Fol* microconidia. Then roots were washed by vigorous shaking in water and imaged in a binocular microscope. Arrows indicate adhering fungal mycelium visible as white filaments covering the roots. $n_{i.ex.}$ = 3, with 4 replicates each. Scale bar, 0.5 cm. Source data from **f** are provided as a Source Data file.

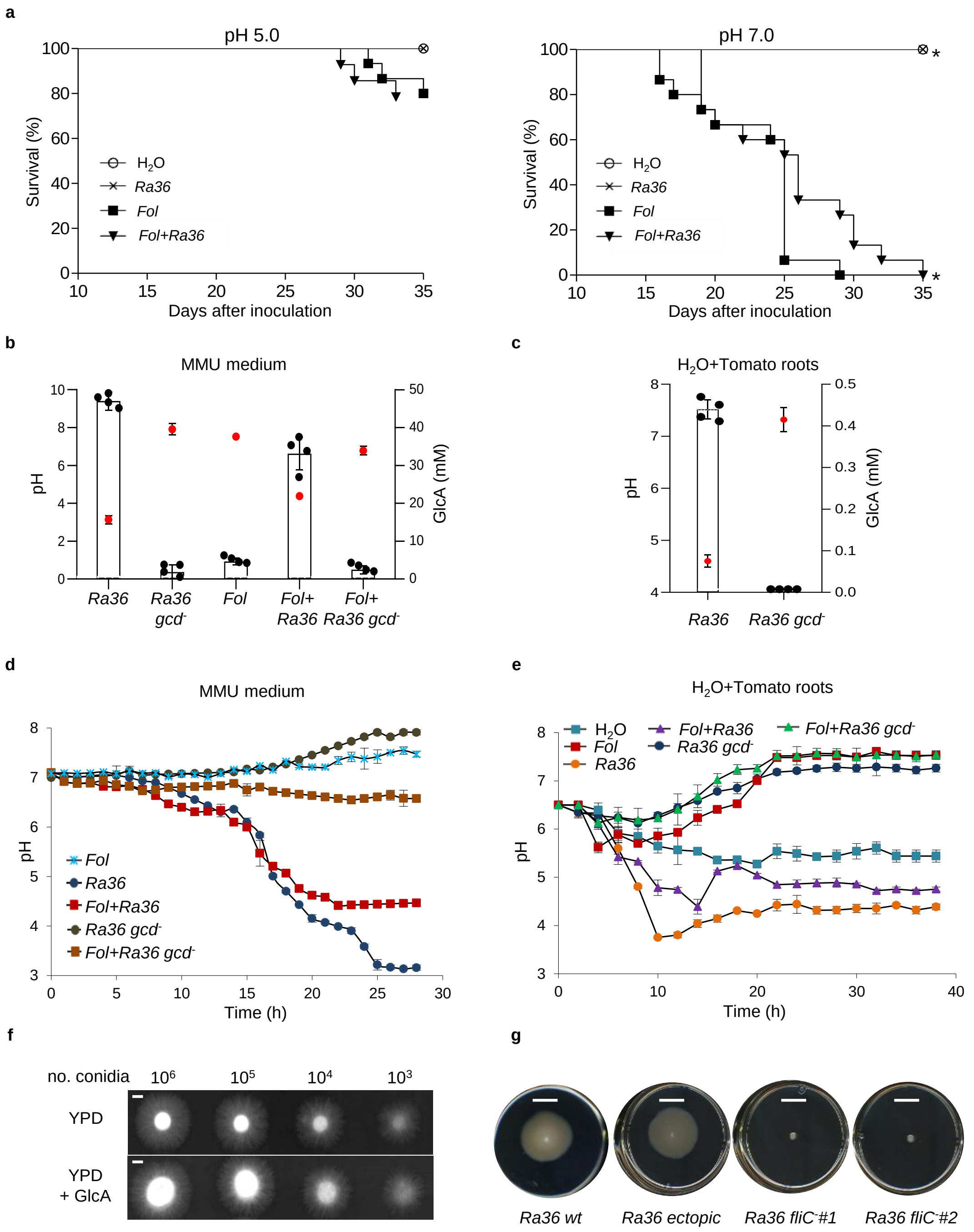

Supplementary Figure 2. Glucose dehydrogenase-mediated production of gluconic acid is required for extracellular acidification by *R. aquatilis*. a. High pH reverts the protective effect of *Ra36* against *Fol* infection on tomato plants. Kaplan–Meier plots showing the survival of tomato plants grown in vermiculite, either uninoculated (H_2O) or dip-inoculated with *Ra36*, *Fol* or both and watered with a solution of 1 mM MES adjusted to pH 5.0 (left) or 7.0 (right). Number of independent experiments ($n_{i.ex.}$). = 3, with 15 plants per treatment. Data shown are from one representative experiment. *$P < 0.05$ versus *Fol* alone according to log-rank test. **b, c.** pH (red dots) and gluconic acid (GlcA, bars) were measured in filter-sterilized supernatants from MMU cultures (**b**) or water containing a submerged tomato root (**c**), at 28h or 72 h, respectively, after inoculation with the indicated *Ra36* strain, *Fol* or both. $n_{i.ex.}$ = 4, with 3 replicates each. Data are presented as mean values +/- SD from three independent experiments. **d, e.** Dynamics of pH was determined in liquid MMU medium (**d**) or water containing a submerged tomato root (**e**) at the indicated time points after inoculation or not (H_2O) with the indicated *Ra36* strain, *Fol* or both. $n_{i.ex.}$ = 3, with 3 replicates each. Data are presented as mean values +/- SD from three independent experiments. **f.** Serial dilutions of *Fol* microconidia were spotted on YPD plates in the absence or presence of 0.4 mM GlcA. Plates were incubated 3 d at 28°C before imaging. $n_{i.ex.}$ = 3, with 3 plates each. Scale bar, 1 cm. **g.** Bacterial swimming zones were imaged 24 h after spot-inoculation of the indicated *Ra36* strains on 0.3% LB agar plates. *Ra36 fliC-* #1 and #2 correspond to independent isogenic *fliC-* deletion mutants. Representative plates are shown. $n_{i.ex.}$ = 3; 4 plates each. Scale bar, 2 cm. Source data from **a-e** are provided as a Source Data file.

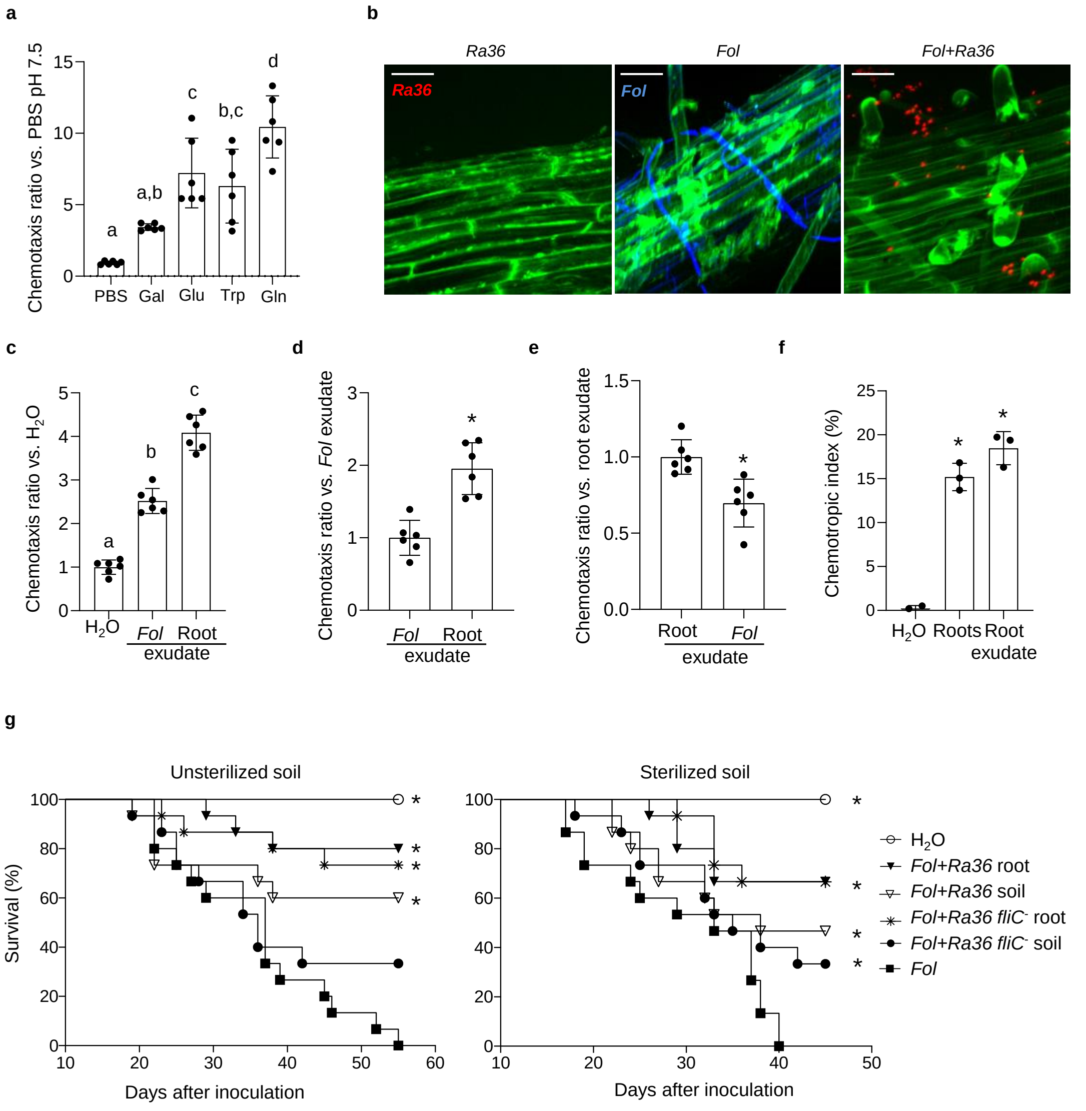

Supplementary Figure 3. *R. aquatilis* exhibits chemotaxis towards *Fol* hyphae and tomato root exudate. a. Chemotaxis capillary assay of *Ra36* maintained in PBS adjusted to pH 7.5, towards gradients of galactose (Gal), glucose (Gluc), tryptophan (Trp) or glutamine (Gln). Number of independent experiments ($n_{i.ex.}$) = 6, with 3 replicates each. Columns with the same letter are not significantly different according to one-way ANOVA followed by Tukey's multiple comparison test ($P < 0.05$). Data are presented as mean values +/- SD from six independent experiments. **b**. Fluorescence microscopy showing maximum projections (50 Z-sections) of representative tomato roots stained with calcofluor white (false colour green), 48 h after inoculation with RFP-tagged *Ra36* (red) and/or GFP-tagged *Fol* (false colour blue) in the experimental set-up shown in Fig. 4b. $n_{i.ex.}$ = 3, with 2 replicates each. Scale bar, 50 µm. **c-e.** Chemotaxis capillary assay of *Ra36* maintained in water and exposed to competing gradients of *Fol* and root exudates (**c**); maintained in *Fol* exudate and exposed to tomato root exudate (**d**); or maintained in tomato root exudate and exposed to *Fol* exudate (**e**). $n_{i.ex.}$ = 6, with 3 replicates each. Data are presented as mean values +/- SD from six independent experiments. *$P < 0.05$ versus *Fol* (**d**) or root exudate (**e**) according to two-tailed, unpaired Student's t-test. In **c**, columns with the same letter are not significantly different according to one-way ANOVA followed by Tukey's multiple comparison test ($P < 0.05$). **f**. Directed growth of *Fol* germ tubes inoculated with *Ra36* towards tomato roots or tomato root exudate. $n_{i.ex}$ = 3, with 5 independent batches of cells per treatment; n = 100 cells per batch. *$P < 0.05$ versus H_2O according to Yates' corrected Chi-squared test. Data are presented as mean values +/- SD from three independent experiments. **g**. Kaplan-Meier plots showing survival of tomato plants grown in unsterilized (left) or sterilized (right) horticultural soil and inoculated or not (H_2O) with the indicated microorganism(s), either mixed with the soil (soil) or dip-inoculated onto roots (root). $n_{i.ex.}$ = 3, with 15 plants per treatment. Data shown are from one representative experiment. *$P < 0.05$ versus *Fol* alone according to log-rank test. Source data from **a, c-g** are provided as a Source Data file.

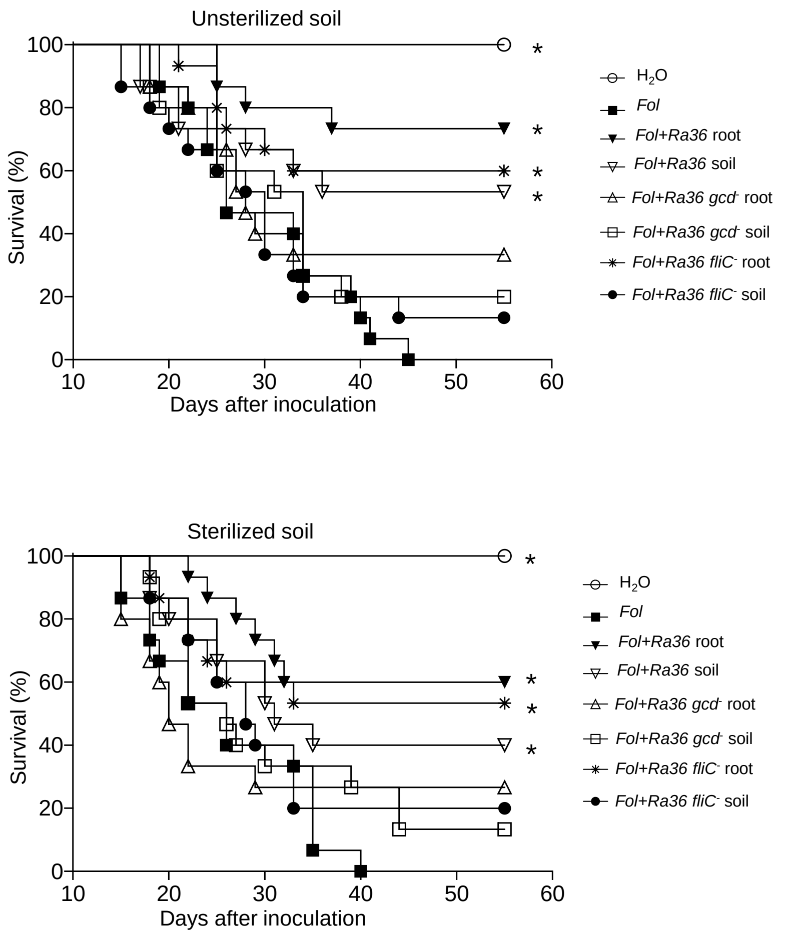

Supplementary Figure 4. Results of biological replicates of key virulence assays. Biological replicates of the experiments shown in Fig. 1f and Supplementary Fig. 3g. Kaplan-Meier plots showing survival of tomato plants grown in unsterilized (upper) or sterilized (lower) horticultural soil and inoculated or not (H_2O) with the indicated microorganism(s), either mixed with the soil (soil) or dip-inoculated onto roots (root). $n_{i.ex.} = 3$, with 15 plants per treatment. *$P < 0.05$ versus *Fol* alone according to log-rank test. Source data are provided as a Source Data file.
