# Supplementary material for: A bacterial endophyte exploits chemotropism of a fungal pathogen for plant colonization
Source: Nat Commun. 2020 Oct 16;11:5264. doi: 10.1038/s41467-020-18994-5 (PMC7567819; doi:10.1038/s41467-020-18994-5)
Supplement: Supplementary file 3 — Description of Additional Supplementary Files [file 41467_2020_18994_MOESM3_ESM.pdf]

Description of Additional Supplementary Files

File Name: Supplementary Movie 1
Description: Movement of *R. aquatilis* within the tomato root xylem. Time-lapse microscopy of *Ra36* motility in the root xylem of a tomato seedling under bright-field optics. Tomato roots were observed with a Zeiss Axio Imager M2 Dual Cam epifluorescence microscope (Zeiss) and a 40X objective. Time is given in hours:min:sec (Observation: 190 frames, 15 frames per second). Scale bar, 20 μm. Refers to Supplementary Fig. 1d.

File Name: Supplementary Movie 2
Description: Movement of *R. aquatilis* within the tomato root xylem. Time-lapse microscopy of RFP-tagged (false colour white) Ra36 cells in the root xylem of a tomato seedling under fluorescence optics. The tomato root was observed with a Zeiss Axio Imager M2 Dual Cam epifluorescence microscope (Zeiss) and a 40X objective. Time is given in hours:min:sec (Observation: 190 frames, 15 frames per second). Scale bar, 20 μm. Refers to Supplementary Fig. 1d.

File Name: Supplementary Movie 3
Description: Movement of *R. aquatilis* in the film of liquid surrounding *F. oxysporum* hyphae. Time-lapse microscopy of *Ra36* motility along a *Fol* hyphae observed under bright-field optics with a Zeiss Axio Imager M2 Dual Cam epifluorescence microscope (Zeiss) and a 40X objective. Note that *Ra36* cells accumulate at the hyphal tip. Time is given in hours:min:sec (Observation: 305 frames, 15 frames per second). Scale bar, 20 μm. Refers to Fig. 4a.

File Name: Supplementary Movie 4
Description: Movement of *R. aquatilis* in the film of liquid surrounding *F. oxysporum* hyphae. Time-lapse microscopy of RFP-tagged (false colour white) *Ra36* cells moving along a *Fol* hyphae observed under fluorescence optics with a Zeiss Axio Imager M2 Dual Cam epifluorescence microscope (Zeiss) and a 40X objective. Note that *Ra36* cells accumulate at the hyphal tip. Time is given in hours:min:sec (Observation: 170 frames, 15 frames per second). Scale bar, 20 μm. Refers to Fig. 4a.

File Name: Supplementary Movie 5
Description: Movement of *R. aquatilis* along hyphae of *F. oxysporum* before contacting a tomato root. Movement of RFP-tagged (false colour white; contrast inverted) Ra36 cells along *Fol* hyphae was observed under fluorescence optics with a SteREO Lumar.V12 fluorescence stereomicroscope (Zeiss). Note that *Ra36* cells accumulate along the tips of leading *Fol* hyphae. Scale bar, 100 μm. (Observation: 1 min 16 sec, 154 frames, 15 frames per second). Refers to Fig. 4c-d.

File Name: Supplementary Movie 6
Description: *R. aquatilis* moving from *F. oxysporum* hyphae to the tomato rhizoplane. Movement of RFP-tagged (false colour white; contrast inverted) *Ra36* cells was observed under fluorescence optics with a SteREO Lumar.V12 fluorescence stereomicroscope (Zeiss). Note that *Ra36* cells move from the tips of leading *Fol* hyphae into the liquid film (indicated by a light blue line) surrounding the edges of a tomato root (indicated by green lines) and

accumulate on the root surface (arrows). Scale bar, 100 µm. (Observation: 2 min 29 sec, 300 frames, 15 frames per second). Refers to Fig. 4c-d.

File Name: Supplementary Data 1
Description: *P* values and statistical tests used to analyze experimental datasets.
