# Supplementary material for: A bacterial endophyte exploits chemotropism of a fungal pathogen for plant colonization
Source: Nat Commun. 2020 Oct 16;11:5264. doi: 10.1038/s41467-020-18994-5 (PMC7567819; doi:10.1038/s41467-020-18994-5)
Supplement: Supplementary file 11 — Reporting Summary [file 41467_2020_18994_MOESM11_ESM.pdf]

nature research

Corresponding author(s): Antonio di Pietro and David Turrà

Last updated by author(s): Sep 3, 2020

Reporting Summary

Nature Research wishes to improve the reproducibility of the work that we publish. This form provides structure for consistency and transparency in reporting. For further information on Nature Research policies, see our Editorial Policies and the Editorial Policy Checklist.

Statistics

For all statistical analyses, confirm that the following items are present in the figure legend, table legend, main text, or Methods section.

n/a	Confirmed	
☐	☒	The exact sample size (n) for each experimental group/condition, given as a discrete number and unit of measurement
☐	☒	A statement on whether measurements were taken from distinct samples or whether the same sample was measured repeatedly
☐	☒	The statistical test(s) used AND whether they are one- or two-sided<br>*Only common tests should be described solely by name; describe more complex techniques in the Methods section.*
☒	☐	A description of all covariates tested
☒	☐	A description of any assumptions or corrections, such as tests of normality and adjustment for multiple comparisons
☐	☒	A full description of the statistical parameters including central tendency (e.g. means) or other basic estimates (e.g. regression coefficient) AND variation (e.g. standard deviation) or associated estimates of uncertainty (e.g. confidence intervals)
☐	☒	For null hypothesis testing, the test statistic (e.g. F, t, r) with confidence intervals, effect sizes, degrees of freedom and P value noted<br>*Give P values as exact values whenever suitable.*
☒	☐	For Bayesian analysis, information on the choice of priors and Markov chain Monte Carlo settings
☒	☐	For hierarchical and complex designs, identification of the appropriate level for tests and full reporting of outcomes
☒	☐	Estimates of effect sizes (e.g. Cohen's d, Pearson's r), indicating how they were calculated

Our web collection on statistics for biologists contains articles on many of the points above.

Software and code

Policy information about availability of computer code

Data collection	As stated in the Methods section (page 15): Images were captured and processed using Axiovision 4.8, ZEN lite 2.3 (both from Zeiss) or ImageJ (v1.52).
Data analysis	As stated in the Methods section (page 8): Identification of gcd and fliC gene orthologs from different R. aquatilis strains was performed with the BLAST algorithm39, using the R. aquatilis HX2 gcd and fliC genes as baits. Nucleotide sequences encompassing the entire gcd and fliC genes and comprising 1.5-kb upstream and downstream of the retrieved ORFs were aligned using the BioEdit 7.0.0 software (Ibis Bioscience, Carlsbad, CA, USA).<br>As stated in the Methods section (page 15): Images were captured and processed using the Axiovision 4.8, ZEN lite 2.3 (both from Zeiss) or ImageJ (v1.52).<br>As stated in the Methods section (page 15): Statistical analyses were performed using GraphPad Prism 5 software (GraphPad Software).

For manuscripts utilizing custom algorithms or software that are central to the research but not yet described in published literature, software must be made available to editors and reviewers. We strongly encourage code deposition in a community repository (e.g. GitHub). See the Nature Research guidelines for submitting code & software for further information.

Data

Policy information about availability of data

All manuscripts must include a data availability statement. This statement should provide the following information, where applicable:

- Accession codes, unique identifiers, or web links for publicly available datasets
- A list of figures that have associated raw data
- A description of any restrictions on data availability

The R. aquatilis HX2 gcd and fliC genes (GenBank A.N.: “EF090904 [https://www.ncbi.nlm.nih.gov/nuccore/EF090904]” and “AFE58723 [https://

www.ncbi.nlm.nih.gov/protein/AFE58723]", respectively) were used to design primers for the amplification of the gcd and fliC gene orthologs from different R. aquatilis strains. Sequence data are deposited in the European Nucleotide Archive under accession number "LT708303 [https://www.ebi.ac.uk/ena/browser/view/LT708303]" and in Genbank under accession number "MN972473 [https://www.ncbi.nlm.nih.gov/nuccore/MN972473]", respectively. The data underlying Figures 1a,b,f; 2d-e; 3b-e; 4e; Supplementary Figures 1a,f; 2a-e; 3a,c-g and 4 are provided as source data file. The statistical tests used to analyze each experimental dataset as well as all the obtained significances and exact P values are provided in the Supplementary data 1 file. All other data are available from the corresponding author upon reasonable request.

Field-specific reporting

Please select the one below that is the best fit for your research. If you are not sure, read the appropriate sections before making your selection.

- [x] Life sciences
- [ ] Behavioural & social sciences
- [ ] Ecological, evolutionary & environmental sciences

For a reference copy of the document with all sections, see nature.com/documents/nr-reporting-summary-flat.pdf

Life sciences study design

All studies must disclose on these points even when the disclosure is negative.

Sample size	The number of replicates for each experiment is provided. In all cases we used three to six biological replicates for experiments as that is standard practice for most genetic or microbiological assays (see Turra et al. 2015 Nature; Masachis et al .2016 Nat Microbiol; Vitale et al. 2019 Nat Microbiol) and to ensure reproducibility of the presented results.
Data exclusions	No data was excluded.
Replication	All experimental findings reported in the paper were reliably reproduced during three to six independently replicated experiments. Additionally, all key experiments were also independently performed by two different researchers.
Randomization	Fungal strains and tested conditions were allocated to groups randomly.
Blinding	No blinding was used as processing procedures and the application of specific treatments made blinding not possible.

Reporting for specific materials, systems and methods

We require information from authors about some types of materials, experimental systems and methods used in many studies. Here, indicate whether each material, system or method listed is relevant to your study. If you are not sure if a list item applies to your research, read the appropriate section before selecting a response.

Materials & experimental systems

n/a	Involved in the study	
[x]	[ ]	Antibodies
[x]	[ ]	Eukaryotic cell lines
[x]	[ ]	Palaeontology and archaeology
[x]	[ ]	Animals and other organisms
[x]	[ ]	Human research participants
[x]	[ ]	Clinical data
[x]	[ ]	Dual use research of concern

Methods

n/a	Involved in the study	
[x]	[ ]	ChIP-seq
[x]	[ ]	Flow cytometry
[x]	[ ]	MRI-based neuroimaging
